# Transcriptional regulation of amino acid metabolism in response to nitrogen deficiency and nitrogen forms in tea plant root (*Camellia sinensis* L.)

**DOI:** 10.1038/s41598-020-63835-6

**Published:** 2020-04-22

**Authors:** Tianyuan Yang, Huiping Li, Yuling Tai, Chunxia Dong, Xunmin Cheng, Enhua Xia, Ziping Chen, Fang Li, Xiaochun Wan, Zhaoliang Zhang

**Affiliations:** State Key Laboratory of Tea Biology and Utilization, Anhui Agricultural University, Hefei, China

**Keywords:** Molecular biology, Plant sciences

## Abstract

Free amino acids, including theanine, glutamine and glutamate, contribute greatly to the pleasant taste and multiple health benefits of tea. Amino acids in tea plants are mainly synthesized in roots and transported to new shoots, which are significantly affected by nitrogen (N) level and forms. However, the regulatory amino acid metabolism genes have not been systemically identified in tea plants. Here, we investigated the dynamic changes of free amino acid contents in response to N deficiency and forms in tea plant roots, and systemically identified the genes associated amino acid contents in individual metabolism pathways. Our results showed that glutamate-derived amino acids are the most dynamic in response to various forms of N and N deficiency. We then performed transcriptomic analyses of roots treated with N deficiency and various forms of N, and differentially expressed amino acid metabolic genes in each pathway were identified. The analyses on expression patterns and transcriptional responses of metabolic genes to N treatments provided novel insights for the molecular basis of high accumulation of theanine in tea plant root. These analyses also identified potential regulatory genes in dynamic amino acid metabolism in tea plant root. Furthermore, our findings indicated that the dynamic expression levels of *CsGDH, CsAlaDC, CsAspAT, CsSDH, CsPAL, CsSHMT* were highly correlated with changes of amino acid contents in their corresponding pathways. Herein, this study provides comprehensive insights into transcriptional regulation of amino acid metabolism in response to nitrogen deficiency and nitrogen forms in tea plant root.

## Introduction

Tea is one of the most popular nonalcoholic beverages in the world. It is consumed daily by billions of people worldwide for its attractive taste and significant health benefits, which are conferred by the high abundance of polyphenols, caffeine, and amino acids^1–3^. Free amino acids account for 1–5% dry weight in tea leaves of green teas. Among these amino acids, theanine (Thea), glutamine (Gln), glutamic acid (Glu) and arginine (Arg) are the most abundant^4,5^. Characteristically, Thea is a unique non-protein amino acid in tea plant (*Camellia sinensis* L.), it can account for more than 70% of total free amino acids and up to 2% of the dry weight of leaves^6–8^. The health benefits of Thea include induction of relaxation, anti-paralysis induced by caffeine, anti-tumor, anti-obesity and body weight control. These benefits have been extensively studied and reported by more than 500 research articles and nearly 300 review papers^9^. Free amino acids also contribute to the formation of tea aroma compounds and a large number of other secondary metabolites essential for tea plant growth and stress adaption^10,11^. However, the molecular mechanism of amino acid metabolism regulation in tea plant is still poorly understood.

**Figure 1:**
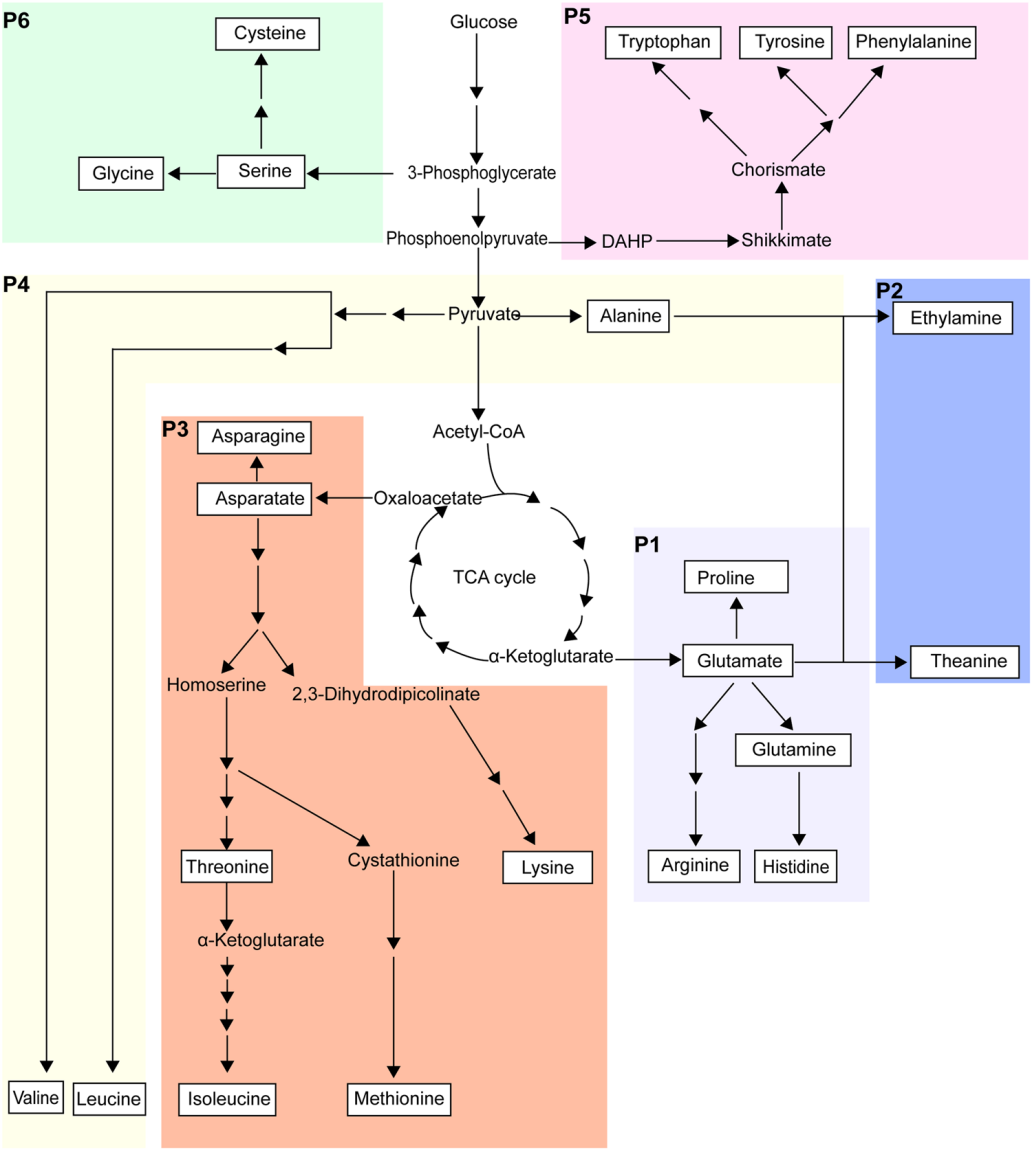
Schematic diagram of amino acid metabolism pathways in tea plants. P1&2, amino acids were derived from Glu pathway consisting of Glu, Gln, Arg, Pro and Thea. P3, amino acids were derived from Asp pathway consisting of Asp, Thr, Ile, Met, Lys, Asn. P4, amino acids were derived from pyruvate pathway consisting of Val and Leu. P5, amino acids were derived from aromatic amino acid pathway consisting of Trp, Phe and Tyr. P6, amino acids were derived from 3-phosphoglycerate pathway consisting of Cys, Ser and Gly. Glu, glutamate; Gln, glutamine; Arg, arginine; Pro, proline; Thea, theanine; Asp, aspartate; Thr, threonine; Lys, lysine; Ile, isoleucine; Aspn, asparagine; Val, Valine; Leu, Leucine; Trp, tryptophan; Phe, Phenylalanine; Tyr, Tyrosine; Cys, cysteine; Gly, Glycine; Ser, serine.

In plants, amino acids are synthesized through branched pathways^12,13^ (Fig. 1). 2-oxoglutarate provides carbon skeleton for Glu, Gln, proline (Pro) and Arg biosynthesis. Oxaloacetate is the initial metabolite for synthesis of asparagine (Asp), aspartate (Asn), threonine (Thr), lysine (Lys), methionine (Met), and isoleucine (Ile). Alanine (Ala), leucine (Leu) and valine (Val) are synthesized from pyruvate. Aromatic amino acids tryptophan (Trp), tyrosine (Tyr) and phenylalanine (Phe) are the products of the shikimate pathway. 3-phosphoglycerate is the substrate of serine (Ser), glycine (Gly) and cysteine (Cys) synthesis. In tea plant, Thea is synthesized from Glu and ethylamine (EA) by theanine synthetase (TS)^14^. EA is likely produced from alanine under the catalysis of alanine decarboxylase^15^.

Amino acid catabolism have been clearly described in animals, however, limited information of amino acid catabolism is available in plants. Hildebrandt *et al*. summarized the catabolic pathways of amino acids in land plants. Generally, amino acids are catabolized by oxidative deamination, oxidative decarboxylation, and transamination. These reactions are catalyzed by amino acid dehydrolases, decarboxylases and aminotransferases, respectively. Gln, Asn and Arg are also hydrolyzed by asparaginase, glutaminase, and arginase to release amide groups^16^.

The accumulation of free amino acids is resultant of biosynthesis and catabolism. Previous studies showed that feedback inhibition loops control amino acid biosynthesis in plants^17^. Here, the accumulation of an amino acid inhibits the transcription or the activities of the enzymes in its biosynthesis pathway^18–20^. Expression of feedback-insensitive form of enzymes resulted in higher levels of the corresponding amino acids^17,21^. The central role of amino acid catabolism is to adjust the amino acid pool size, especially under stress conditions^12^. Some regulatory enzymes in amino acid metabolism have been identified in model plants. However, studies have demonstrated that amino acid metabolism is regulated by a large number of general and specific factors, and the regulation differs significantly between species, tissues, developmental stages, various stresses and stages of stress responses^12,22,23^. Members of genes encoding isoforms of enzymes catalyzing a specific step in amino acid metabolism also usually play different roles in these processes^24,25^.

In tea plants (*Camellia sinensis* L.), amino acid metabolism is affected by nitrogen (N) levels and forms, and environmental factors^26–30^. Tea plants prefer to uptake and use NH_4_^+ ^^26,31,32^. Basically, application of N fertilizers increases amino acid biosynthesis in tea plant. When equimolar concentrations of NO_3_^−^ and NH_4_^+^ were supplied, NH_4_^+^ more efficiently promoted tea plant growth and amino acid accumulation^26,27,33,34^. In addition, intensive studies showed that shading treatment significantly increases free amino acid accumulation in tea plant^29,35,36^. The alteration of amino acid metabolism under various conditions was suggested to be associated with gene expression and activity of glutamine synthetase (GS), glutamate synthase (GOGAT), glutamate dehydrogenase (GDH) and other amino acid biosynthesis genes^26,34,36^. However, only a few genes involved in amino acid metabolism and genes associated with changes in amino acid accumulation have been identified in tea plants.

As the most abundant free amino acid in tea plant, Thea was first discovered by Sakato^37^. Thea metabolism has been studied for more than 60 years, but its molecular mechanism remains largely unknown. It has been reported that TS catalyzes the biosynthesis of Thea from Glu and EA^14^ (Fig. 1). The gene encoding TS was recently identified in tea plant^38^. However, Cheng *et al*.^39^ showed that GSs from tea plants and other plants, such as *Arabidopsis*, also have the capacity to synthesize Thea. They further speculated that high accumulation of EA is why tea plant can synthesize large amount Thea. EA was suggested to be synthesized from alanine under the catalysis of alanine decarboxylase. In the other hand, Thea could be degraded into EA and Glu by theanine hydrolase^40^. Until now, the gene encoding for theanine hydrolase has not been identified yet. Finally, it is noteworthy that Thea is mainly synthesized in roots and is transported through the vascular system to tea plant shoots^41–45^.

The complete sequencing of the tea plant genome now provides a means to systematically identify genes encoding enzymes in individual amino acid metabolic steps^38,46^. In this study, we cultured tea plants under N free condition or with the supply of different forms of N (NO_3_^−^-N, EA-N, NH_4_^+^-N, and [NH_4_^+^ + NO_3_^−^]-N) to achieve significantly different accumulation patterns of free amino acids in the roots of these tea plants. The responses to N forms of free amino acid production in each synthesis pathway were analyzed. The corresponding genes in amino acid metabolic pathways were identified, and the expression patterns of these genes were characterized in roots by RNA-seq analyses. These analyses have identified fundamental and regulatory mechanisms of amino acid metabolism in tea plant.

## Results

### Glu pathway amino acids are most abundant and most dynamic in response to N level and N forms in tea plant roots

**Figure 2:**
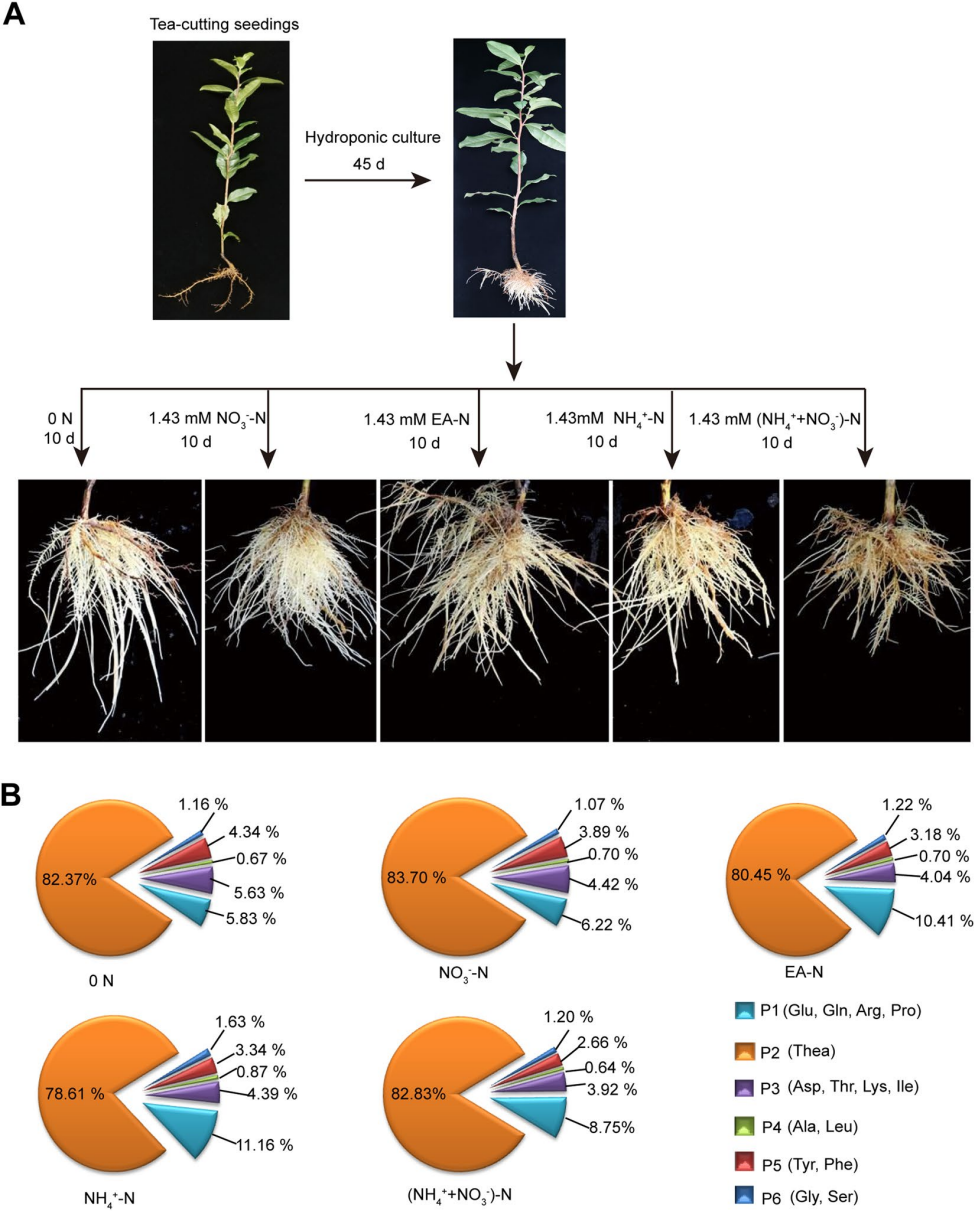
Schematic of experiment procedure and composition of amino acids in the tea plant roots under the treated conditions. (**A**) Two-year-old cuttings of tea plant were recorded after hydroponics cultivation for 45d in a basal nutrient solution. The roots morphologies were recorded under various N forms treatment for 10 d (see “Materials and Methods”). (**B**) Composition of amino acids in tea plant roots under different forms of N and 0 N treatments at time point of 10 d.

To study the regulation of amino acid metabolism in tea plant roots, we hydroponically cultured tea plants to produce well developed roots (Fig. 2A). These plants were then treated with equal concentrations of N in the forms of Ca(NO_3_)_2_ (NO_3_^−^-N), ethylamine hydrochloride (EA-N), (NH_4_)_2_SO_4_ (NH_4_^+^-N), or (NH_4_)_2_SO_4_ + Ca(NO_3_)_2_ ([NH_4_^+^ + NO_3_^−^]-N), along with a nitrogen free (0 N) control. After 10 days, tea plants under these treatments developed varied root architecture system (Fig. 2A). Given that root architecture is responsive to N status for better nutrient foraging^47,48^ and is associated with amino acid levels^49^, this result suggested there were differences in the endogenous amino acid contents in these tea plant roots.

**Figure 3:**
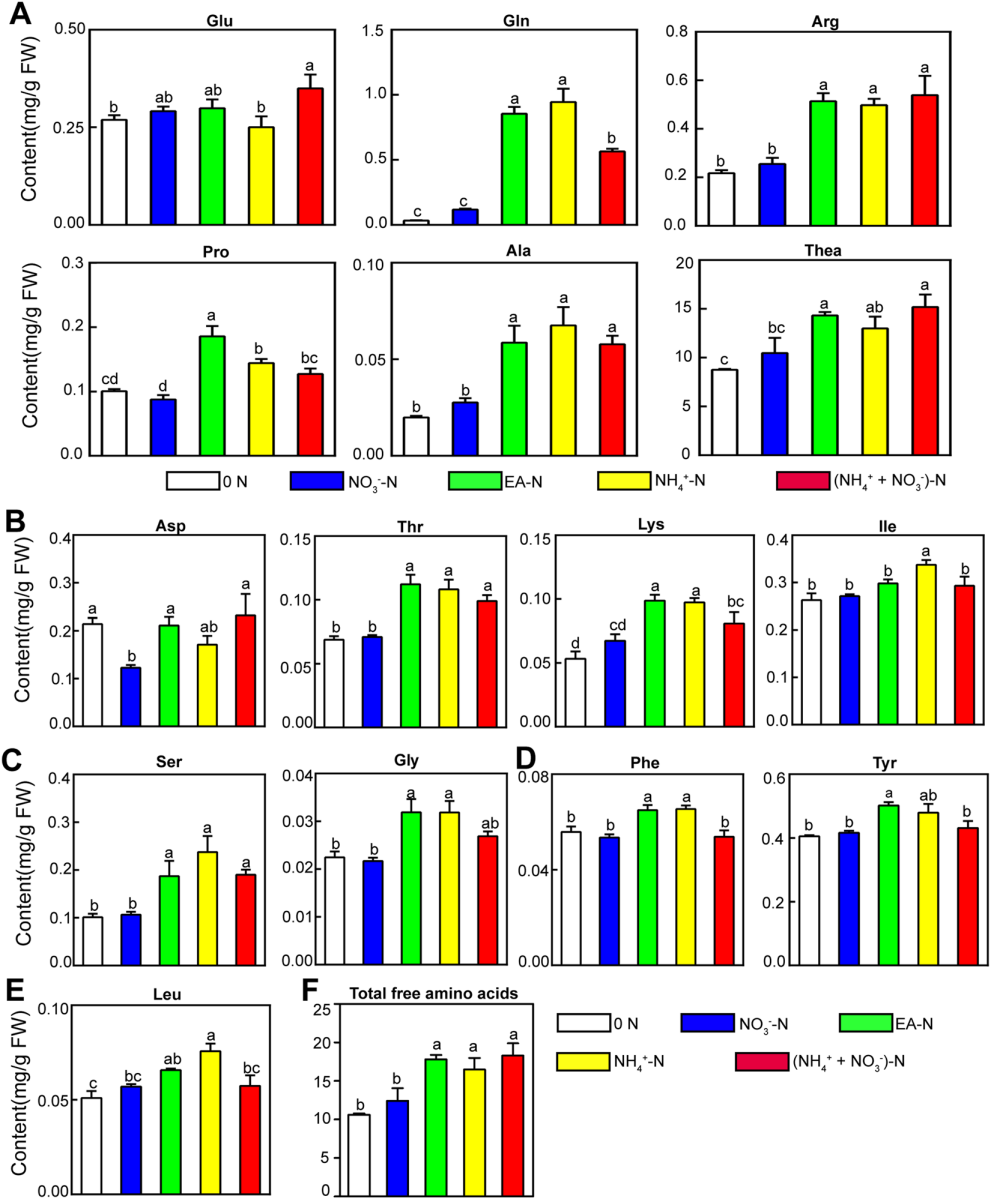
Effects of N forms and 0N on accumulation of amino acids in tea plant roots. The contents of 15 amino acids were determined in tea plant roots under various forms of N and 0 N treatments. Ala: Alanine; Ser: Serine; Gln: Glutamine; Pro: Proline; His: Histidine; Gly: Glycine; Arg: Arginine; Thr: Threonine; Lys: Lysine; Tyr: Tyrosine; Thea: Theanine; Leu: Leucine; Phe: Phenylalanine; Asp: Aspartic acid; Ile: Isoleucine; Glu: Glutamic acid. Data shown are the average mean ± SE of three replicates (n  =  3). Different letters indicate statistical significance among different treatments according to Duncan’s multiple range test at the 5% level.

The contents of main amino acids derived from Glu pathway (Glu, Gln, Arg, Pro, and Thea), Asp pathway (Asp, Ile, Thr, Lys), pyruvate pathway (Ala and Leu), aromatic amino acid pathway (Phe and Tyr) and 3-phosphoglycerate pathway (Ser and Gly) were measured under the treated conditions. The results showed that Glu pathway amino acids accounted for ~90% of the total free amino acids examined in tea plant roots (Fig. 2B, Table S1). Among these amino acids examined, theanine content was the highest and reached over 1.5% fresh weight and 73.6%-83.7% of the total free amino acids examined, followed by Gln, Arg, Glu, and Tyr contents (Figs. 2B, 3, Table S1). Contents of Ile, Asp, Ser, and Pro were similar, and the contents of Ala, Leu, Thr, Lys, Gly and Phe were low in tea plant roots under the treated conditions.

How the free amino acid accumulation changed in response to 0 N and various forms of N were then examined. Generally, the contents of total free amino acids were similar under 0 N and NO_3_^−^-N (Fig. 3A–F). The contents were also similar under the EA-N, NH_4_^+^-N and (NH_4_^+^ + NO_3_^−^)-N, and were ~50% higher under these conditions than under 0 N (Fig. 3A–F, Table S1). This is consistent with the previous observation that NH_4_^+^-N is more efficient at promoting amino acid biosynthesis than NO_3_^−^-N ^31^. Interestingly, Glu contents were stable under these conditions, with only a slight increase under (NH_4_^+^ + NO_3_^−^)-N. Conversely, the contents of Glu-derived amino acids including Gln, Arg, Pro and Thea changed significantly under the different conditions. Impressively, Gln contents were low under 0 N and NO_3_^−^N and were greatly up-regulated (~28 fold) by EA-N and NH_4_^+^-N (Figs. 3A, S1A). In contrast, the accumulation of amino acids in Asp pathway, aromatic amino acid pathway, 3-phosphoglycerate pathway and Lea in pyruvate pathway were less responsive to 0 N and N forms in tea plants under our experimental condition (Figs. 3B–E and S1B-E, Table S1). These results indicate that Glu pathway is not only the main flux of amino acid metabolism, but it is also most responsive to N deficiency and N forms.

### Contents of Thea and Ala responded similarly to 0N and N forms

Thea is synthesized from Glu and EA. Thea contents increased ~50% under EA-N, NH_4_^+^-N and (NH_4_^+^ + NO_3_^−^)-N relative to the 0 N condition (Figs. 3A and S1A, Table S1). The result showed that Glu contents were stable even under 0 N for 10 days. EA is the product of Ala decarboxylation. Contrastingly to the Glu contents, Ala contents significantly changed in accordance with Thea contents. This correlation suggested that formation of EA from Ala may comprise the main regulatory step of Thea synthesis.

Surprisingly, a direct supply of equimolar EA (1.43 mM) did not significantly increase Thea accumulation compared with the NH_4_^+^-N and (NH_4_^+^ + NO_3_^−^)-N treatments. EA-N promoted the accumulation of all amino acids examined as efficiently as NH4^+^-N and (NH_4_^+^ + NO_3_^−^)-N (Fig. 3, Table S1). This implied, when low level of EA as the sole nitrogen source, EA is not used in priority as precursor to synthesize Thea but rather used for the synthesis of all amino acids.

### Metabolism of amino acids derived from the same precursors may be regulated in modules in response to N levels and forms

We further noticed that amino acids from the same pathway showed similar accumulation patterns in response to 0 N and N forms. The contents of Asp-derived Thr and Lys both changed ~1.8 fold from 0 N and NO_3_^−^-N to NH_4_^+^ containing conditions (EA-N, NH_4_^+^-N and [NH_4_^+^ + NO_3_^−^]-N) (Figs. 3B and S1B, Table S1). Meanwhile, the accumulation of 3-phosphoglycerate pathway-derived Ser and Gly also showed similar response patterns. In addition, branched-chain amino acids (Leu and Ile) and aromatic amino acids (Phe and Tyr) showed similar and slight changes (Figs. 3B–E, S1B–E, Table S1). These results demonstrated that metabolism of amino acids in the same pathway is likely regulated as a module, and may be controlled by genes encoding key enzymes catalyzing the common steps.

### An over view of genes expression profiles and identification of DEGs related to amino acid metabolism

To explore the molecular mechanism of amino acid accumulation in response to 0 N and N forms in tea plant roots, total RNA were extracted from roots of tea plants treated with 0 N, NO_3_^−^-N, EA-N, NH_4_^+^-N and (NH_4_^+^ + NO_3_^−^)-N for 10d (Fig. 2A). The total RNA was used to prepare cDNA libraries for transcriptomic analysis. Four biological replicates were performed. Therefore, 20 cDNA libraries were sequenced using the Illumina HiSeq platform. In total, 133.79 Gb of clean reads were generated, with an average of 6.69 Gb of clean reads per sample. Additionally, the Q20 (the percentage of bases with a Phred value >20) value for the clean reads was > 98% and the Q30 (the percentage of bases with a Phred value >30) value of the clean reads was >94% (Table S1), implying high quality sequencing results were obtained for the following analyses. The clean reads were mapped to the reference genome^38^. Approximately 80% of reads were successfully mapped; the uniquely mapped ratio was about 60% (Table S2), indicating that the sequencing qualities of all samples were comparable.

**Figure 4:**
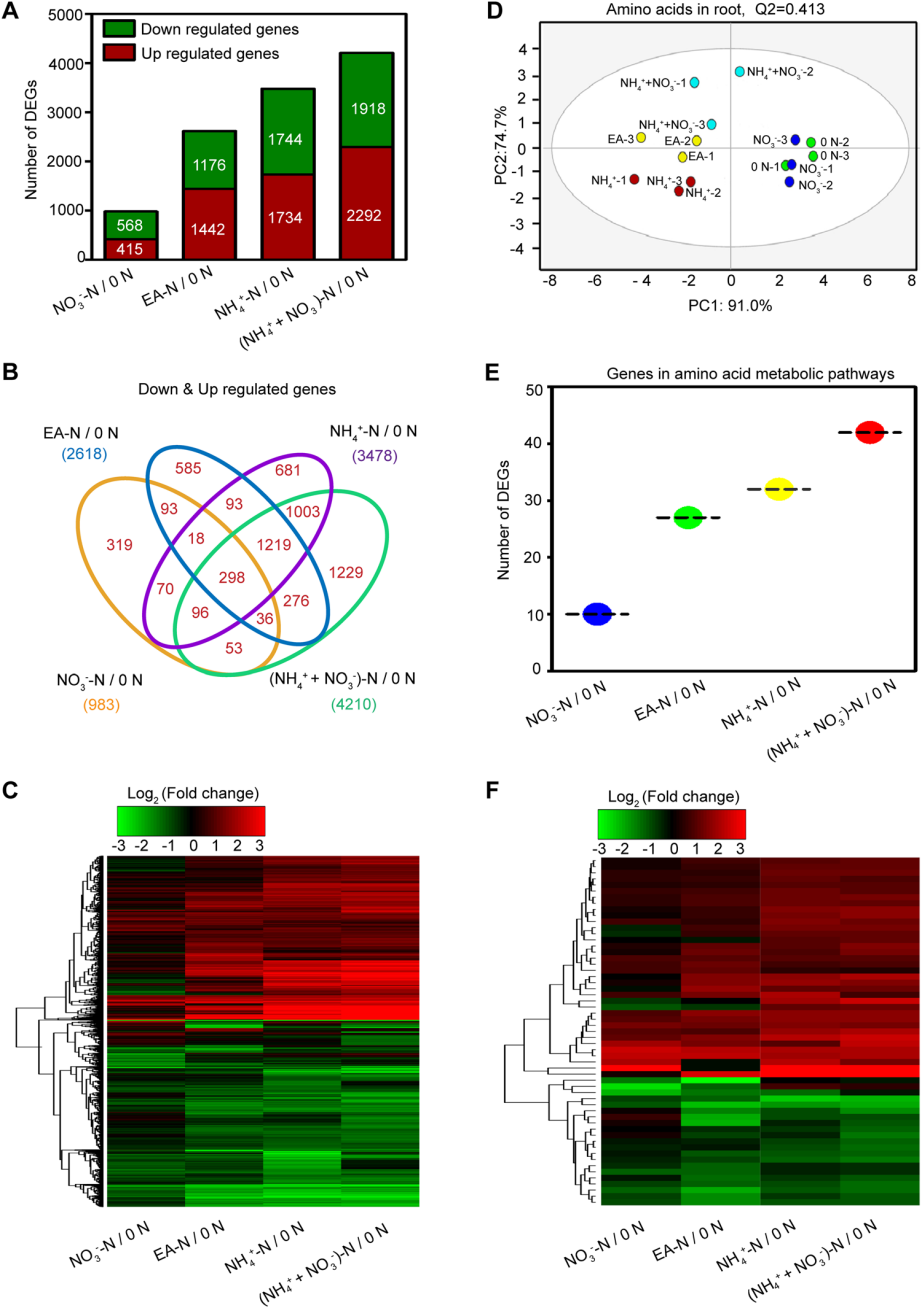
An overview on differentially expressed genes responsive to different forms of N in tea plant root. (**A**) The number of DEGs was examined in the comparisons between 0 N and each form of N in tea root. DEGs, differentially expressed genes. (**B**) Venn diagram showing distribution of DEGs in the comparisons between 0 N and various forms of N treatments. (**C**) Hierarchical clustering represents relative expression levels of DEGs in comparisons between 0 N and various N forms. The FPKM ratio of gene expression is represented on a logarithmic scale for treatments with different N forms ( NO_3_^−^-N, EA-N, NH_4_^+^-N, and [NH_4_^+^ + NO_3_^−^]-N) relative to the control (0 N) (log_2 adj_FPKM_N forms_ /_adj_FPKM_0 N_). _adj_FPKM, adjusted Fragment Per Kilo base of exon model per Million mapped reads. Red indicates a gene up-regulated at that treatment, while green indicates down-regulated expression. (**D**) The OPLS-DA analysis of amino acids in tea plant roots under treatments with different forms of N. Data shown are from the value of three biological replicates (n = 3). OPLS-DA analysis was performed by SIMCA 13.0 (UMETRICS, https://umetrics.com/). (**E**) Numbers of DEGs related to amino acids metabolism were calculated in the comparisons between 0 N and various forms of N treatments. (**F**) Hierarchical clustering representing relative expression levels of DEGs related to amino acids metabolism.

A total of 49073 genes were identified and their expression levels in the roots under the different treatments were measured (Table S3). In order to get insights into how the gene expression in tea plant root responds to N forms, we set 0 N as a control treatment. A total of 6005 DGEs were identified by pairwise comparisons: 0 N vs. NO_3_^−^-N, 0 N vs. EA-N, 0 N vs. NH_4_^+^-N, and 0 N vs. (NH_4_^+^ + NO_3_^−^)-N. The comparisons found 983, 2618, 3478 and 4210 DGEs for these comparisons, respectively (Fig. 4A; Table S4). A Venn diagram was constructed to investigate the numbers of co-expressed and uniquely expressed DEGs in response to different N forms (Fig. 4B; Table S5). A total of 298 co-expressed DEGs were obtained under treatment of all four N forms. In addition, hierarchical clustering analysis showed that strong changes in DEG expression levels were observed in EA-N, NH_4_^+^-N and (NH_4_^+^ + NO_3_^−^)-N treatment conditions (Fig. 4C), whereas slight changes in expression levels of DEGs were found in the NO_3_^−^N treatment. These results agreed with the observed changes in the total number of DEGs for NO_3_^−^-N (Fig. 4A,B).

To examine the effect of different N forms on amino acid accumulation, an OPLS-DA analysis was performed to analyze 15 amino acids in tea roots under those treatments. Amino acid profiling showed that treatments of different N forms and N deficiency affected amino acids accumulation in tea roots (Fig. 4D). The general pattern of amino acid accumulation was consistent with the number of DEGs and DEG expression levels in NO_3_^−^-N, EA-N, NH_4_^+^-N and (NH_4_^+^ + NO_3_^−^)-N treatment conditions.

Subsequently, the DEGs encoding enzymes in amino acid biosynthesis and the first step of amino acid degradation were also identified. Similarly with total numbers of DEGs, the number of DEGs for the NO_3_^−^-N was much less than that for the EA-N, NH_4_^+^-N and (NH_4_^+^ + NO_3_^−^)-N conditions (Fig. 4E). Hierarchical clustering analysis also revealed stronger changes in these DEG expression levels in EA-N, NH_4_^+^-N and (NH_4_^+^ + NO_3_^−^)-N relative to NO_3_^−^-N treatment (Fig. 4F).

### High expression *CsAlaDC*, *CsTSI* and *CsGS* was associated with the abundance and response of Thea to 0 N and N forms in tea plant root

In order to elucidate the molecular basis of amino acid accumulation in response to 0 N and N forms in tea plant root, we systemically identified genome-wide genes encoding biosynthetic enzymes as well as enzymes catalyzing the initial amino acid catabolic steps. A total of 380 genes encoding 75 enzymes were identified and their expression levels were presented in Table S6.

**Figure 5:**
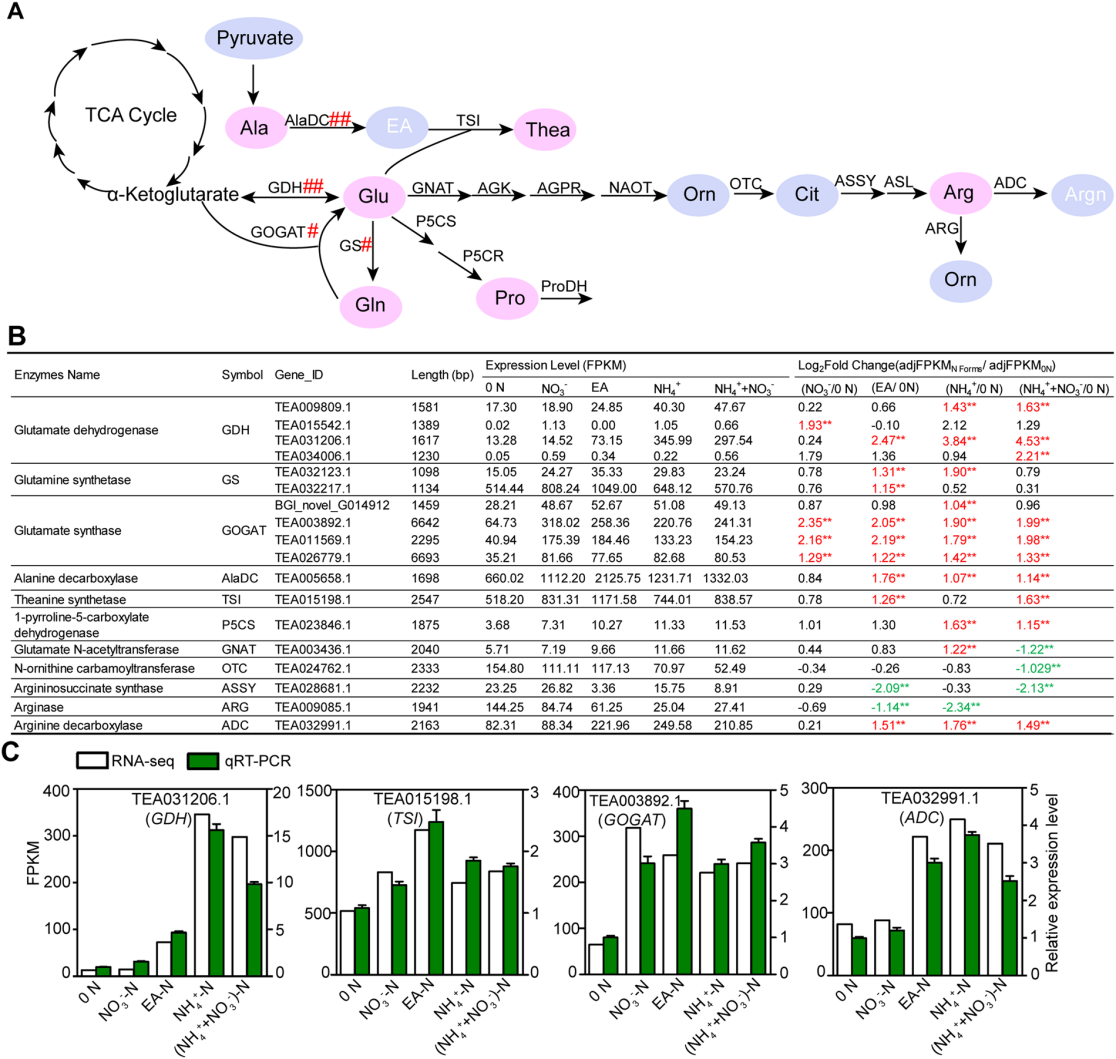
Identification of DEGs encoding enzymes related to Glu pathway. (**A**) The DEGs encoding enzymes related to synthesis and first step degradation of the Glu pathway. (**B**) Expression levels and relative fold change (log_2_ [_adj_FPKM_N forms_/_adj_FPKM_0 N_]) of DEGs related to Glu-derived amino acids. _adj_FPKM, adjusted Fragment Per Kilo base of exon model per Million mapped reads. The table indicates genes with significant changes (fold change ≥ 2, *p* < 0.05; marked by two asterisks and number in red or green) in 0 N versus different N forms. 0 N, N free; NO_3_^−^, NO_3_^−^-N, EA, Ethylamine-N; NH_4_^+^, NH_4_^+^-N; (NH_4_^++^NO_3_^−^), (NH_4_^+^ + NO_3_^−^)-N. (**C**) Quantitative real-time PCR validation for potential candidate genes. The relative expression levels and FPKM values are shown.

As shown in Fig. 2B, Thea was the most abundant free amino acid in the roots. The two precursors of Thea synthesis are EA and Glu which are produced by CsAlaDC, CsGDHs and CsGOGATs, respectively (Fig. 5A). EA and Glu are catalyzed by CsCsTSI or CsGSs to synthesize Thea. The (NH_4_^+^ + NO_3_^−^)-N is normally used in tea hydroponic culture^50^. Under this treatment, within the 316 amino acid biosynthetic genes, *CsAlaDC*, *CsCsTSI*, *CsGS* (TEA032217.1) ranked the top 3 most highly expressed genes (Table S6). Impressively, total FPKM of these 3 genes accounted for 25.78% of the total FPKM of all the 316 genes. Furthermore, total FPKM of *CsAlaDC*, *CsGDHs*, *CsGOGATs*, *CsCsTSI* and *CsGSs* accounted for as high as 38.65% of the total FPKM of all these 316 genes. Therefore, the high expression of these Thea-related genes provide strong basis for the highly abundant accumulation of Thea in tea plant roots.

Comparing with other Thea-related amino acid biosynthetic genes, *CsAlaDC* was more associated with Thea abundance and response to 0 N and N forms. *CsAlaDC* was not only the 1^st^ most highly expressed amino acid synthetic gene (Table S6), it was also the 5^th^ most highly expression genes within all genes in tea plant roots under EA-N condition (Table S3). More importantly, *CsAlaDC* expression was induced by NH_4_^+^ containing treatments (EA-N, NH_4_^+^-N, [NH_4_^+^ + NO_3_^−^]-N) (Fig. 5B, Table S7), and showed a similar pattern as Thea accumulation (Fig. 3A). Although *CsTSI* and *CsGS* (TEA032217.1) were also the 2^nd^ and 3^rd^ most highly expressed amino acid synthetic genes, their expression was relatively stable and was only induced by EA-N (Fig. 5B). These results suggested *CsAlaDC* plays more regulatory role in Thea biosynthesis.

### *CsGOGATs* and Arg catabolic genes responded distinctively to 0 N and N forms from other amino acid metabolic genes in Glu pathway

Glu is the initial product of ammonia assimilation and provides α-amino group for all other amino acid biosynthesis. It also provides carbon skeleton for Pro abd Arg biosynthesis. Therefore, Glu plays a central role in amino acid metabolism in plants^51^. In this study, Glu contents kept stable in tea roots under 0 N, NO_3_^−^-N, EA-N, NH_4_^+^-N and (NH_4_^+^ + NO_3_^−^)-N conditions (Fig. 3A). This probably was the resultant of reduced biosynthesis and promoted catabolism of other amino acids under 0 N. Consistently, we observed that 0 N down-regulated the expression of amino acid synthetic genes, and up-regulated the expression amino acid catabolic genes (Fig. 5B, Table S7).

Except for Glu, the contents of Gln, Pro and Arg were significantly lower under 0 N and NO_3_^−^N condition than that in EA-N, NH_4_^+^-N and (NH_4_^+^ + NO_3_^−^)-N conditions (Fig. 3A). Consistently, most of the differently expressed amino acid synthetic genes in Glu pathway were up-regulated by EA-N, NH_4_^+^-N and (NH_4_^+^ + NO_3_^−^)-N (Fig. 5B). Distinctively, three out of 4 differently expressed *CsGOGATs* were similarly up-regulated by NO_3_^−^-N as well as by EA-N, NH_4_^+^-N and (NH_4_^+^ + NO_3_^−^)-N. This up-regulation of *CsGOGATs* may contribute to the maintenance of Glu content under the supply of NO_3_^−^-N which cannot be efficiently used by tea plants^52^.

Arg can be hydrolyzed by arginase into urea and ornithine (Orn) and was finally degraded into ammonium and carbon dioxide. Alternatively, Arg can also be decarboxlated by Arginine decarboxylase (CsADC) and was further metabolized into polyamines. Interestingly, these two processes were differently regulated by 0 N and N forms. Here, *CsADC* was significantly induced by EA-N, NH_4_^+^-N and (NH_4_^+^ + NO_3_^−^)-N; whereas, *CsARG* was greatly induced by 0 N (Fig. 5B). These results suggested CsADC regulates Arg catabolism into polyamines under N sufficient condition, and CsARG mediates Arg catabolism to ammonium under N deficient condition.

To validate the expression profiles of DEGs obtained from RNA-seq dataset, five DEGs related to the Glu pathway were selected for qRT-PCR, including *CsGDH* (TEA031206.1), *CsTSI*, *CsGOGAT* (TEA003892.1) and *CsADC* (TEA032991.1). The results of qRT-PCR in each treatment closely corresponded to the transcript levels of the RNA-seq dataset (Fig. 5C).

### Expression of *CsAspAT*, *CsAK* and *CsTHS* was responsive to 0 N and N forms in tea plant root

**Figure 6:**
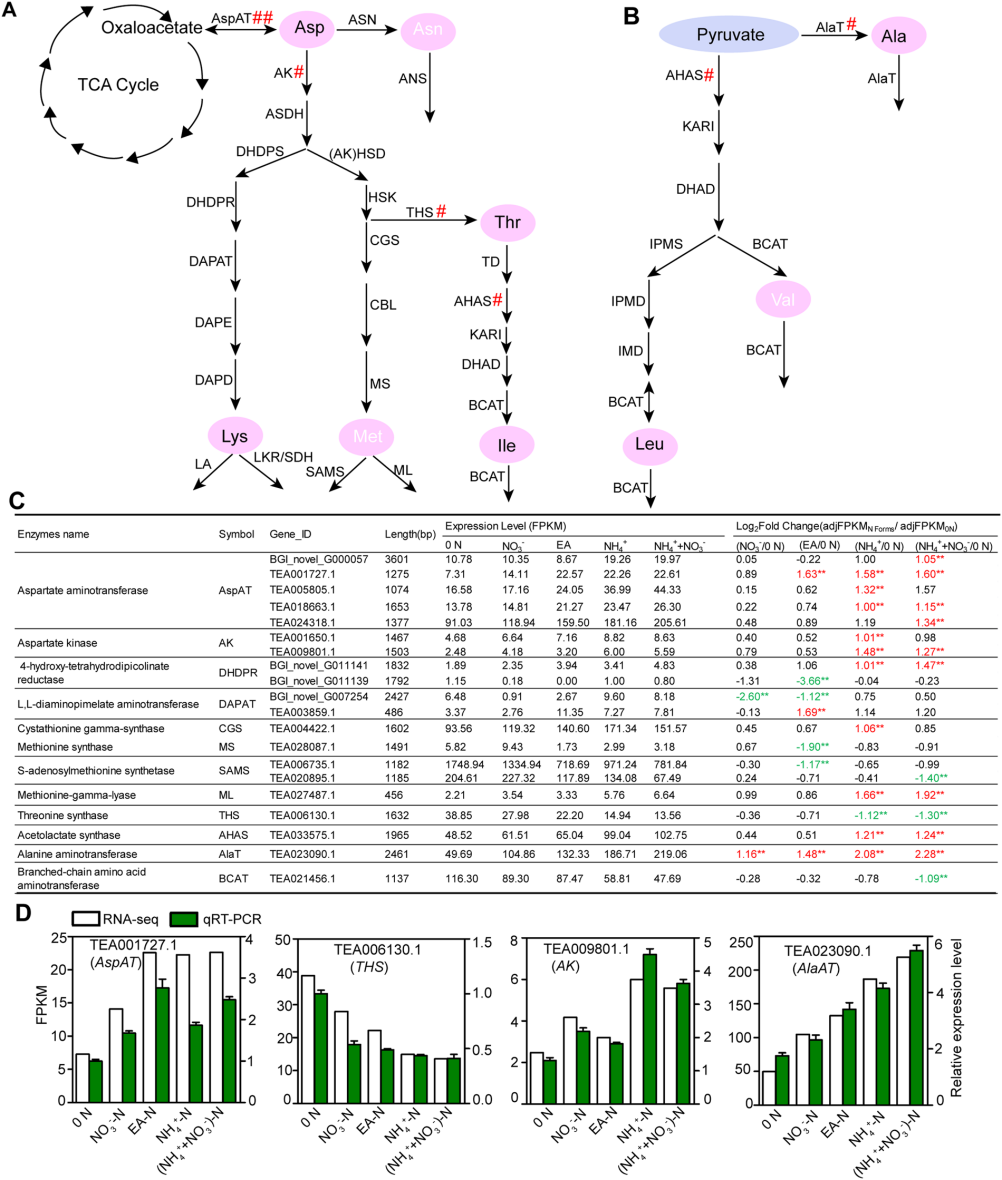
Identification of DEGs encoding enzymes related to Asp and pyruvate pathway. (**A**) The DEGs encoding enzymes related to synthesis and first step degradation pathway of Asp and pyruvate-derived amino acids. (**B**) Expression levels and relative fold change (log_2_ (_adj_FPKM_N forms_/_adj_FPKM_0 N_) of DEGs related to Asp-derived and pyruvate amino acids. The table indicates genes with significant changes (fold change ≥ 2, *p* < 0.05; marked by two asterisks and number in red or green) in 0 N versus different N forms. 0 N, N free; NO_3_^−^, NO_3_^−^-N, EA, Ethylamine-N; NH_4_^+^, NH_4_^+^-N; (NH_4_^++^NO_3_^−^), (NH_4_^+^ + NO_3_^−^)-N. (**C**) Quantitative real-time PCR validation for potential candidate genes. The relative expression levels and FPKM values are shown.

Asp is synthesized from 2-oxaloacetate and Glu under the catalysis of aspartate aminotransferase (AspAT) (Fig. 6A). Asp can then act as precursor to produce Thr, Met, Lys and Ile which are essential for mammals^19^. In this pathway, some 120 genes encoding 25 amino acid metabolic enzymes were identified from transcriptome datasets (Fig. 6A and B; Table S8). Among these 120 genes, nineteen genes were differentially expressed in response to 0 N and N forms (Fig. 6B, Table S8). Especially, genes encoding CsAspAT, asparate kinase (CsAK) and threonine synthase (CsTHS) were more responsive to 0 N and N forms, suggesting important regulatory roles of these genes in Asp pathway.

In this study, we observed that Asp contents in tea plant roots were generally stable under the treatments (Fig. 3B). Under these conditions, five genes encoding CsAspAT and two genes encoding CsAK were significantly upregulated by EA-N, NH_4_^+^-N and (NH_4_^+^ + NO_3_^−^)-N, comparing with the 0 N treatment. These genes were also induced by NO_3_^−^-N, although were not statistically significant (Fig. 6B). These results suggested the Asp biosynthesis was regulated by CsAspAT under 0 N and various N forms. In addition, CsAK catalyses the first step in the conversion of Asp to Lys, Thr, Ile and Met (Fig. 6A). Therefore, reduced expression of *CsAK* under 0 N suggested CsAK regulated the conversion under this condition. Taken together, the stable levels of Asp under the 0 N and the supply with various forms of N were probably coordinated by the expression of *CsAspATs* and *CsAKs*.

Threonine synthase (THS) catalyzes Thr synthesis (Fig. 6A). *CsTHS* (TEA006130.1) expression was higher under 0 N and NO_3_^−^-N conditions (Fig. 6B). However, Thr levels under 0 N and NO_3_^−^-N were lower than under EA-N, NH_4_^+^-N and (NH_4_^+^ + NO_3_^−^)-N conditions (Fig. 3B). These results suggested *CsTHS* expression is probably feedback regulated by Thr accumulation in tea plant roots.

### *CsAHAS* and *CsBCAT* were associated with the branched-chain amino acid Leu and Ile metabolism in response to 0 N and N forms in tea plant root

Although Ile and Leu are derived from Asp and pyruvate, respectively, they are both branched-chain amino acids and share common metabolic enzymes including acetolactate synthase (AHAS), ketol-acid reductoisomerase (KARI), dihydroxy-acid dehydratase (DHAD) and branched-chain amino acid aminotransferase (BCAT) (Fig. 6A,B). We showed that Leu and Ile levels were just slightly responded to 0 N and N forms in the roots; but they showed similar response pattern (Fig. 3B,E). Consistently, within 40 genes encoding 8 enzymes in Ile and Leu metabolism, only *CsAHAS* (TEA033575.1) was significantly up-regulated by NH_4_^+^-N and (NH_4_^+^ + NO_3_^−^)-N, and only *CsBCAT* (TEA021456.1) was significantly down-regulated by (NH_4_^+^ + NO_3_^−^)-N (Fig. 6C, Table S8). These results suggested a regulatory role of *CsAHAS* and *CsBCAT* in branched-chain amino acid metabolism in response to 0 N and N forms in tea plant root.

Ala is synthesized from pyruvate by Alanine aminotransferase (AlaT) (Fig. 6B). *CsAlaT* (TEA023090.1) was significantly induced by EA-N, NH_4_^+^-N and (NH_4_^+^ + NO_3_^−^)-N (Fig. 6C), similarly as the up-regulation of Ala accumulation by these N forms (Fig. 3A). These results suggested an importantly role of *CsAlaT* in Ala biosynthesis.

Four representative genes (*CsAspAT*, *CsTHS*, *CsAK*, *CsAlaT*) were selected for qRT-PCR analysis. Transcript levels determined by qRT-PCR were perfectly matched with those of the RNA-seq dataset (Fig. 6D).

### Multiple regulatory sites and divergent regulatory mode of shikimate pathway in response to 0 N and N forms

The aromatic amino acids (AAA) Phe, Tyr, and Trp are not only essential components of protein synthesis, but also provide the precursors for the synthesis of a wide range of secondary metabolites in plants^53^. The aromatic amino acids are synthesized via the shikimate pathway, which initiates from phosphoenolpyruvate (PEP) and erythrose 4-phosphate (E-4P). The regulation of AAA biosynthesis via the shikimate pathways has been largely unknown in tea plant. Therefore, it is important to characterize the number and expression levels of these genes encoding enzymes leading to shikimate pathway in response to 0 N and different forms of N.

**Figure 7:**
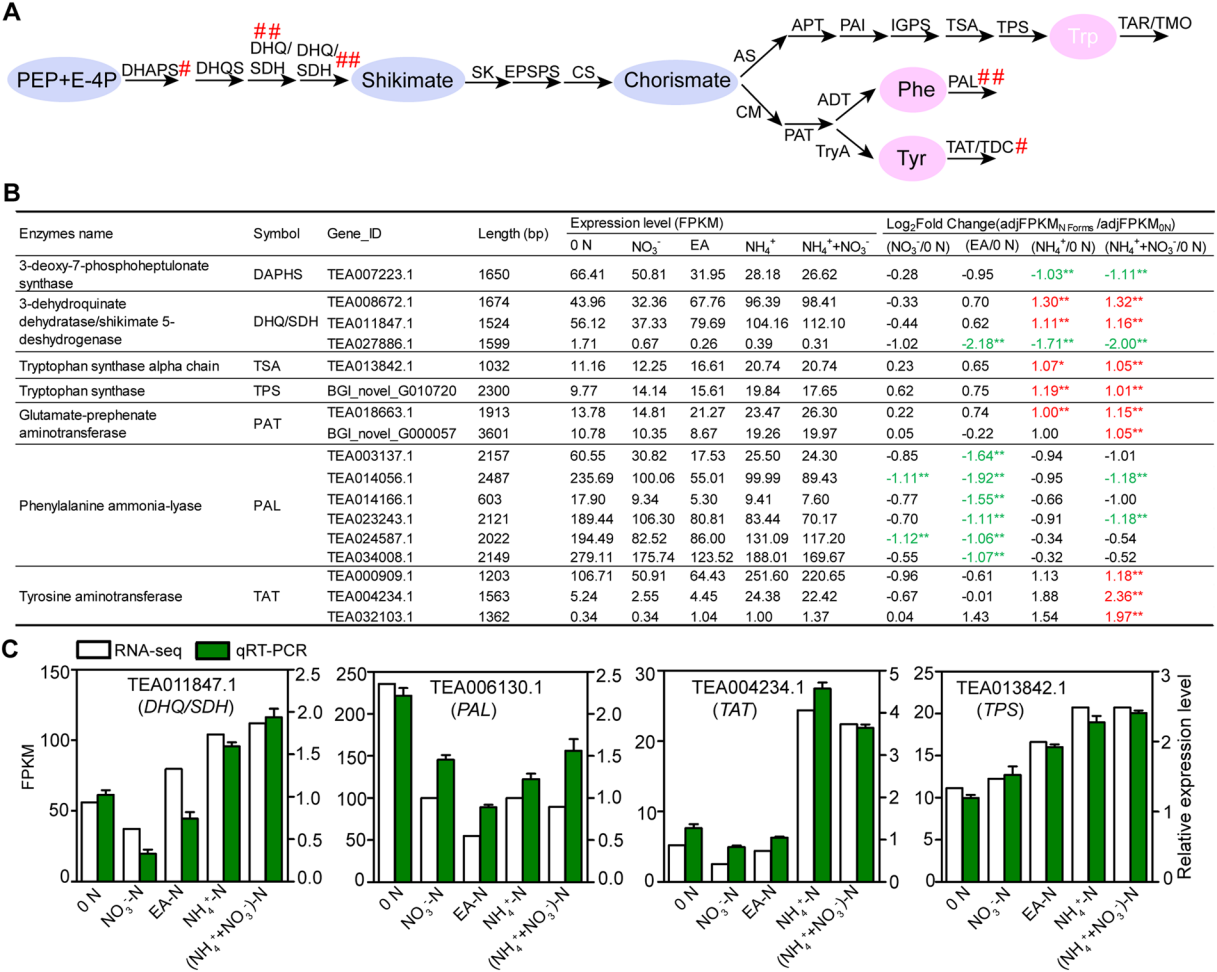
Identification of DEGs encoding enzymes related to phosphoenolpyruvate/shikimate pathway. (**A**) The DEGs encoding enzymes related to synthesis and first step degradation pathway of amino acids from phosphoenolpyruvate/shikimate pathway. (**B**) The expression levels and relative fold change (log_2_ [_adj_FPKM_N forms_/_adj_FPKM_0 N_]) of DEGs related to amino acids from phosphoenolpyruvate/shikimate pathway. The table indicates genes with significant changes (fold change ≥ 2, *p* < 0.05; marked by two asterisks and number in red or green) in 0 N versus different N forms. 0 N, N free; NO_3_^−^, NO_3_^−^-N, EA, Ethylamine-N; NH_4_^+^, NH_4_^+^-N; (NH_4_^+^ + NO_3_^−^), (NH_4_^+^ + NO_3_^−^)-N. (**C**) Quantitative real-time PCR validation for potential candidate genes. The relative expression levels and FPKM values are shown.

In total, 92 annotated genes encoding 19 major enzymes in the shikimate pathway were identified (Fig. 7A,B; Table S9). Among these 92 genes, 17 genes were identified to be DEGs in response to 0 N and N forms (Fig. 7B).

The initial step of shikimate pathway is the formation of 3-dehydroquaianate from PEP and E-4P and this reaction is catalyzed by 3-deoxy-d-arabino-heptulosonate-7- phosphate synthase (DAHPS). Within 5 genes encoding CsDAHPS, one gene (TEA007223.1) was significantly repressed by NH_4_^+^-N and (NH_4_^+^ + NO_3_^−^)-N (Fig. 7B). This result suggested, as the first enzyme of shikimate pathway, CsDAHPS is negatively regulated by NH_4_^+^-N and (NH_4_^+^ + NO_3_^−^)-N at the transcriptional level.

Phe and Tyr levels were increased by ~20% by both EA-N and NH_4_^+^-N (Fig. 7B). However, EA-N did not induce the expression of genes encoding biosynthetic enzymes in shikimate pathways (Fig. 7B, Table S9). Characteristically, EA-N significantly repressed the expression of 6 genes encoding Phenylalanine ammonia-lyase (PAL). NO_3_^−^-N, NH_4_^+^-N and (NH_4_^+^ + NO_3_^−^)-N also repressed the expression of *CsPALs*, but the repression was much weaker than EA-N (Fig. 7B). Phe is a precursor for a large number of important secondary metabolites, including phenylpropanoids, flavonoids, lignin, anthocyanins, catechins, and many other metabolites^53^. The first step of Phe catabolism towards these metabolites is catalyzed by PAL. These results suggested N, especially EA-N, represses Phe catabolism through regulating the expression of *CsPALs*.

Different from EA-N, NH_4_^+^-N up-regulated 2 genes encoding biosynthetic enzymes including 3-dehydroquinate dehydratase/shikimate 5-dehydrogenase (DHQ/SDH), and 1 gene encoding prephenate aminotransferase (PAT) (Fig. 7B). DHQ/SDH catalyses last two step of shikimate synthesis, and PAT catalyses last step of arogenate synthesis^53^. Shikimate is a critical precursor for aromatic amino acid synthesis. Arogenate also serves as a common substrate for both Phe and Tyr synthesis. Therefore, these results suggested NH_4_^+^-N promotes Phe and Tyr synthesis mainly through up-regualting *CsDHQ/SDH* and *CsPAT* expression in tea plant root.

(NH_4_^+^ + NO_3_^−^)-N did not alter Phe and Tyr levels comparing with 0 N and NO_3_^−^-N (Fig. 3D), but it regulated the expression of many genes encoding enzymes in shikimate pathway (Fig. 7B, Table S9). (NH_4_^+^ + NO_3_^−^)-N significantly up-regulated the expression of 2 *CsDHQ/SDHs*, two *CsPATs*, two *CsPALs*, and 3 genes encoding Tyrosine aminotransferase (TAT). TAT catalyzes the first step of Tyr degradation. In addition, as described above, (NH_4_^+^ + NO_3_^−^)-N also repressed the expression of gene encoding CsDAHPS, the first enzyme of shikimate pathway. Thus, (NH_4_^+^ + NO_3_^−^)-N coordinately regulated the expression of genes encoding important biosynthetic and catabolic enzymes in shikimate pathway.

Finally, it is noteworthy that 3 *CsDHQ/SDHs* were identified as DEGs in response to 0 N and N forms, with 2 *CsDHQ/SDHs* were upregulated by NH_4_^+^-N and (NH_4_^+^ + NO_3_^−^)-N and 1 *CsDHQ/SDH* was down-regulated by EA-N, NH_4_^+^-N and (NH_4_^+^ + NO_3_^−^)-N (Fig. 7B). These results suggested varied roles of 3 *CsDHQ/SDHs* in shikimate pathway in response to 0 N and N forms.

To further validate our results, three important genes (*CsPAL*, *CsTAT* and *CsTPS*) were chosen for qRT-PCR analysis. The expression levels of these genes using qRT-PCR were in good accordance with corresponding transcript levels of the RNA-seq dataset (Fig. 7C).

### Systematic identification and expression analysis of genes encoding enzymes related to 3-Phosphoglycerate pathway

**Figure 8:**
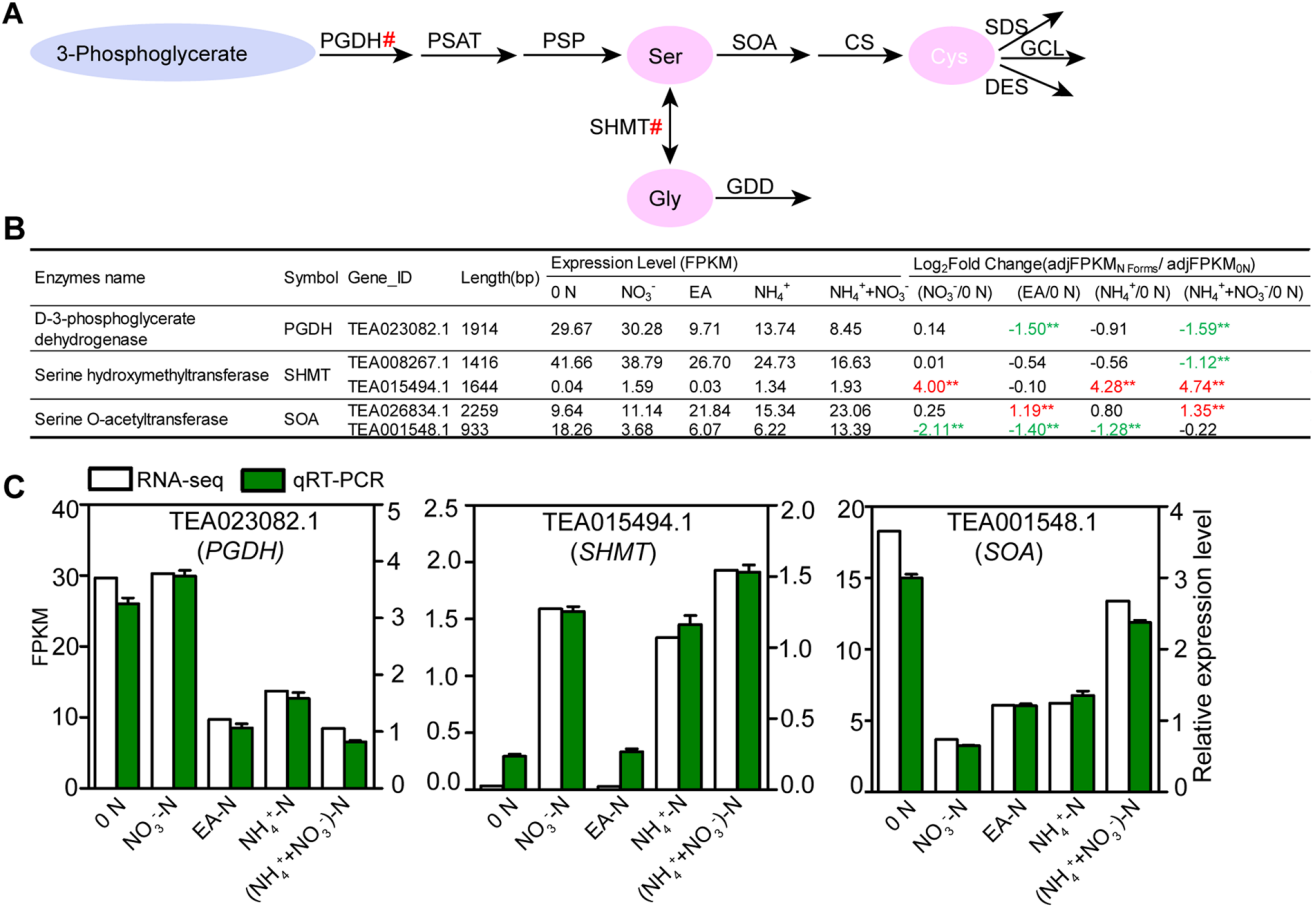
Identification of DEGs encoding enzymes related to 3-phosphoglycerate pathway. (**A**) The DEGs encoding enzymes related to synthesis and first step degradation pathway of amino acids from 3-phosphoglycerate pathway. (**B**) The expression levels and relative fold change (log_2_ [_adj_FPKM_N forms_/_adj_FPKM_0 N_]) of DEGs related to amino acids from 3-phosphoglycerate pathway. The table indicates genes with significant changes (fold change ≥ 2, *p* < 0.05; marked by two asterisks and number in red or green) in 0 N versus different forms of N. 0 N, N free; NO_3_^−^, NO_3_^−^-N, EA, Ethylamine-N; NH_4_^+^, NH_4_^+^-N; (NH_4_^++^NO_3_^−^), (NH_4_^+^ + NO_3_^−^)-N. (**C**) Quantitative real-time PCR validation for potential candidate genes. The relative expression levels and FPKM values are shown.

It was documented that Gly, Cys, and Ser are derived from 3-phosphoglycerate in plants, and are synthesized through 6 reactions catalyzed by 6 enzymes. Genes encoding biosynthetic and catabolic enzymes involved in 3-Phosphoglycerate pathway were screened. In total, 77 annotated genes encoding 10 major enzymes in 3-phosphoglycerate pathways were identified (Fig. 8A; Table S10). Notably, only three DEGs encoding d-3-phosphoglycerate dehydrogenase (*CsPGDH*), Serine hydroxymethyltransferase (*CsSHMT*) and Serine O-acetyltransferase (*CsSOA*) were observed under various forms of N treatments. The transcript abundance of *CsPGDH* was significantly decreased under EA-N and (NH_4_^+^ + NO_3_^−^)-N treatments. Importantly, both *CsSHMT* and *CsSOA* have two members in tea plant, and these showed differential responses to N treatments. The gene expression of *CsSHMT* (TEA008267.1) showed significantly down regulation with (NH_4_^+^ + NO_3_^−^)-N treatment, whereas the gene of *CsSHMT* TEA015494.1 displayed strong induction under NO_3_^−^-N, NH_4_^+^-N and (NH_4_^+^ + NO_3_^−^)-N conditions, suggesting that they might play different roles in response to N forms and levels. Likewise, the gene expression of *CsSOA* (TEA026834.1) was significantly up-regulated under EA-N, (NH_4_^+^ + NO_3_^−^)-N conditions. While, a significant decrease of transcript levels of *CsSOA* (TEA001548.1) was found under NO_3_^−^-N, EA-N and NH_4_^+^-N, but not (NH_4_^+^ + NO_3_^−^)-N conditions (Fig. 8B). These results suggested reverse regulatory roles of 2 *CsSHMTs* and 2 *CsSOAs* in shikimate pathway in response to 0 N and N forms.

To further validate our results, three important genes (*CsPGDH*, *CsSHMT* and *CsSOA*) were chosen for qRT-PCR analysis. The expression levels of these genes using qRT-PCR were consistent with corresponding transcript levels of the RNA-seq dataset (Fig. 8C).

## Discussion

In general, the contents of secondary metabolites significantly affect the quality of tea products^54^. Among the various metabolic products, amino acids greatly contribute to the quality of green tea. Previous studies showed that N forms and N level significantly affect amino acid metabolism, thereby modulating amino acid levels in tea roots and shoots. It is important to achieve a comprehensive understanding of the underlying molecular basis of how amino acid biosynthesis and catabolism are regulated at molecular level by N forms in tea plant root. Several studies have explored amino acid contents and corresponding molecular changes that occur in tea plants in response to nutritional and environmental conditions^26,27,30,43,54–57^.

Our investigation showed that levels of amino acids were significantly regulated by N forms and 0 N. Glu-derived pathway amino acids are most abundant and most dynamic in roots of tea plants. Metabolism of amino acids derived from same precursors may be regulated in modules (Figs. 2, 3). Notably, a direct supply of EA in the culture medium did not increase Thea synthesis, suggesting that Thea might be as a form of nitrogen storage only when N nutrition is sufficient. In present study, we used same amount N concentration as normal nutritional solution. In this condition, the tea plants prefer to utilize EA-N to meet their need for N (Fig. S2), but not directly providing the substrate for Thea synthesis.

Meanwhile, we found that the number and expression levels of DEGs encoding biosynthetic enzymes as well as enzymes that catalyze the first catabolic steps of amino acids were greatly increased by EA-N, NH_4_^+^-N and (NH_4_^+^ + NO_3_^−^)-N compared with those of N deficiency and NO_3_^−^-N treatments (Fig. 4), which is consistent with previous findings^30,55,58^.

Bioavailability of N correlates closely to both tea yield and quality of processed tea^26,27,43^. A broad spectrum of studies have shown that the preference for NO_3_^−^, NH_4_^+^ and mixture of NO_3_^−^ + NH_4_^+^ varies considerably among plant species. For example, maize prefers to utilize NO_3_^−^ nutrient over NH_4_^+^, whereas rice preferentially absorb and assimilate NH_4_^+^ in the roots. Nutrient supplementation level is a critical factor greatly influencing both yield and quality of tea^7,59^. It had been well documented that NH_4_^+^ nutrient is more preferentially and efficiently utilized than NO_3_^−^ nutrient by tea plants and special secondary metabolites are more abundant in supplying with NH_4_^+^ than NO_3_^−^, suggesting that tea plant belongs to NH_4_^+^ preferring plant species^32,52^. This characteristic of NH_4_^+^ preferring was confirmed by ^15^N isotope tracer studies via hydroponically grown tea plants^31,33^. In addition, Ruan *et al*.^32^ reported that the influx rates of NH_4_^+^ were much higher than NO_3_^−^ in tea plant roots. In summary, these findings are consistent with those of this study of amino acids contents in tea roots under various N forms treatments (Fig. 3, Table S1).

Increasing evidences showed that N forms and levels relate closely to changes of amino acids content of tea roots and leaves^26,27,30,41^. However, a comprehensive investigation into the molecular basis underlying amino acids metabolism in tea roots is still absent. For example, Huang *et al*. reported that supplying tea plants with different forms of N significantly increase Pro, Glu, and Thea in tea leaves compared with 0 N, especially when supplied with NH_4_^+^-N^58^, whereas they did not examined amino acid contents changes in tea plant root. Actually, previous studies reported that many amino acids are mainly synthesized in tea root, and are then transported from root to shoot^41,44,45^. Yang *et al*. reported the effects of three N form (NH_4_^+^, NO_3_^−^ and NH_4_^+^ + NO_3_^−^) treatments for 5 min and 96 h on gene expression, but they mixed root, stem, leaf and shoot samples together for the analysis. Thus, the tissue-specific response of gene expression could not be elucidated^30^. Recently, Liu *et al*. reported that short-term (30 min) 10 mM NH_4_^+^-N or NO_3_^−^-N treatment significantly changed the expression of genes in multiple secondary metabolism pathways, and they proposed that NH_4_^+^ and NO_3_^−^ act as signaling agents in regulating gene expression^34^.

Deep RNA-sequence technology is a powerful tool to systemically identify key gene candidates in many plants, such as *Poplar*^60^, *Arabidopsis*^61^, *Camellia sinensis*^30,62,63^. To better understand the mechanism of the changes of amino acids in response to N forms and N deficiency, we examined genes encoding enzymes involved in amino acid biosynthesis and initial steps of catabolism under NO_3_^−^-N, EA-N, NH_4_^+^-N, and NH_4_ + NO_3_-N and N deficiency treatments via deep RNA-seq technology. Based on the analysis of our transcriptome data, the number and gene expression levels of DEGs associated with N metabolism exhibited significantly different under NO_3_^−^-N, EA-N, NH_4_^+^-N, and (NH_4_^+^ + NO_3_^−^) -N in comparison with those of 0 N treatment (Fig. 4). Fewer DEGs were identified in the NO_3_^−^-N treatment, whereas there were more DEGs under EA-N, NH_4_^+^-N and (NH_4_^+^ + NO_3_^−^)-N. This suggested that the genes involved in N absorption, assimilation and metabolism were remarkably affected by the forms of N. Our results partially explain the preference for NH_4_^+^-N at the transcript level (Fig. 4).

Combined with the RNA-seq data, we identified the genes encoding enzymes involved in five main amino acid metabolism pathways. Glu-derived amino acids accounted for more than 90% of total content (Fig. 3, Table S1), suggesting that genes in this pathway and changes in their expression levels greatly contributed to amino acids metabolism in tea plant root. Notably, FPKM of *CsAlaDC*, *CsGDHs*, *CsGOGATs*, *CsCsTSI* and *CsGSs* of Thea-related amino acid biosynthetic genes accounted for as high as 38.65% of the total FPKM of all 316 genes (Tables S5, S6). We speculate that high expression of these genes conferred the highly specific synthesis and accumulation of Thea in tea plant root.

In Asp and pyruvate pathway, aspartate aminotransferase (AspAT) catalyzed 2-oxaloacetate and Glu to synthesize Asp. Asp can be hydrolyzed by asparate kinase (CsAK). As shown in Fig. 6C, the transcript levels of *CsAK* and *CsAspAT* were remarkably responsive to NH_4_^+^-N and (NH_4_^+^ + NO_3_^−^)-N, whereas no significant differences were observed under NO_3_^−^-N and EA-N comparing with 0 N, except for *CsAspAT* (TEA001727.1). These results suggest that the stable levels of Asp were probably caused by responses of *CsAspATs* and *CsAKs* expression under the 0 N and the supply with different N forms.

In addition, Phe is a precursor for many tea secondary metabolites. The first step of Phe catabolism is catalyzed by PAL. Our results showed EA-N significantly represses Phe catabolism by down-regulated of *CsPALs*, suggesting that less metabolism of Phe occurred in this treatment of shikimate pathway. Moreover, due to the significant variation of Ser and Gly contents under different forms of N and levels, we also found a key regulatory DEG (*CsSHMT*) in the 3-Phosphoglycerate pathway, which was significantly responsive to N forms treatment. Interestingly, two *CsSHMTs* and 2 *CsSOAs* displayed contrast gene expression profile under various forms of N and 0 N conditions. This result suggested that there is a reverse regulatory role of 2 *CsSHMTs* and 2 *CsSOAs* in shikimate pathway in response to 0 N and N forms.

We have identified some key regulatory genes in the five main pathways of amino acid metabolism, which provided a vital and useful clue to comprehensively understand the changes of amino acid accumulation in tea roots. However, the molecular mechanism related to how these potential genes control amino acid metabolic flux in tea roots remains unclear. Future studies of these regulatory genes will be needed to further determine the mechanistic effects.

## Conclusion

In this study, integrated transcriptome and metabolites (amino acids) analyses provide new insights into amino acid metabolism of tea roots. The results showed that Glu-derived pathway amino acids are the most abundant and most dynamic in tea roots. Metabolism of amino acids derived from same precursors may be regulated as modules. Moreover, the amino acid composition in tea roots is significantly regulated in response to different forms of N and N deficiency. This study first systematically identified the key potential genes encoding biosynthetic enzymes as well as enzymes catalyzing the initial catabolic steps of amino acids, which can be used for providing a reference and guidance for further research on the role of these potential genes in amino acid metabolism of tea plant roots.

## Materials and methods

### Plant materials and growing conditions

Two-year-old tea cutting seedings (*Camellia sinensis* L. cv. shuchazao) were collected from Dechang Tea Fabrication Base at Shucheng County in Anhui province, China, and used for the hydroponic culture experiments in this study. In the hydroponic experiment, roots of the seedlings collected were washed in tap water to remove the soil on the root surface, and then tea cutting seedlings of similar size with 10–12 leaves were selected and transplanted into plastic pots containing 10 liters of tap water. After 3 days, seedlings were transferred to 5-litre plastic bucket (5 plants per bucket) for hydroponic culture. Basal nutrient solution was supplied stepwise at 1/8 strength of its concentration for 5 days, 1/4 strength for 5 days, and 1/2 strength for another 5 days. Afterwards, the complete basal nutrient solution was supplied for one month. The composition of the nutrient solution was used as described^50^: 0.535 mM (NH_4_)_2_SO_4_, 0.18 mM Ca(NO_3_)_2_, 0.1 mM KH_2_PO_4_, 0.413 mM K_2_SO_4_, 0.392 mM CaCl_2_, 1.029 mM MgSO_4_, 6.27 μM C_10_H_12_FeN_2_NaO_8_, 9.25 μM H_3_BO_3_, 3.9 μM CuSO_4_, 18.2 μM MnSO_4_, 0.4 mM Al_2_(SO_4_)_3_.18H_2_O, 0.53 μM Na_2_MoO_4_ and 1.53 μM ZnSO_4_. The pH of the nutrient solution was adjusted to 4.5. These seedlings were grown in a growth chamber under controlled environmental conditions (light intensity of 200 μmol phtotons m^−2^ s^−1^ for 14 h per day, day/light temperature of 25/22 °C, relative humidity of 70%).

For the nitrogen (N) treatments, basal nutrient solution without N (0 N), or with 0.715 mM Ca(NO_3_)_2_ (1.43 mM NO_3_^−^-N)_,_ 1.43 mM ZtNH_2_.HCl (1.43 mM EA-N), 0.715 mM (NH_4_)_2_SO_4_ (NH_4_^+^-N) and 0.535 mM (NH_4_)_2_SO_4_ + 0.18 mM Ca(NO_3_)_2_ (1.43 mM [NH_4_^+^ + NO_3_^−^]-N), respectively, were used to cultivate tea cuttings seeding. After 10 days of treatments, root samples from each treatment were collected and placed in −80 °C until further use.

### Free amino acids analysis

The determination of free amino acids in tea plant roots was performed as described^64,65^ with minor modifications. Briefly, a HPLC system (Waters 2695) coupled to a fluorescence detector (Waters 2475) and an ultraviolet-visible detector (Waters 2489) was used in this study. We used the Waters AccQ•Tag method with a Waters AccQ•Tag column (Nova-Pak C18, 4 μm,150 mm × 3.9 mm) to examine free amino acids according to the protocol of the AccQ•Fluor Reagent Kit. 10 μL of extraction was injected into the HPLC system for analysis. Thea standard was purchased from Sigma Chemical Company (St. Louis, MO, USA), and other amino acid standards were purchased from Waters Corporation (Milford, Massachusetts, U.S.A).Total contents of free amino acids content were calculated as the sum of each individual free amino acid.

### RNA isolation, Illumina sequencing and data analysis

Total RNA was extracted from root samples using the RNA pure plant Kit (Tiangen, Beijing, China) combined with the improved CTAB method described previously^66^. Agarose gel electrophoresis and NanoDrop 2000 spectrophotometer (Thermo) were used to determine the quality of samples. Libraries were then constructed and sequenced using the Illumina Genome Analyzer (Solexa). All samples for Digital Gene Expression were run in four biological replicates, and each replicate was a mixture of roots from 5 individual tea seedlings. After removing the low quality raw data reads, all remaining high quality clean sequencing reads were mapped onto the tea plant genome reference^38^ to identify continuous gene regions using SOAPaligner/SOAP2, and only two nucleotide mismatches was allowed^67^. Unique mapped reads were used for further analysis.

### Identification of differently expressed genes, functional annotation and classification

The fragments per kilobase of transcript sequence per millions of base pairs sequenced (FPKM) presented the normalized gene expression^68^. The differentially expressed genes (DEGs) among samples with different N treatments were defined using threshold as fold change ≥ 2.00 and adjusted P ≤ 0.05 according to the method^63^. For functional annotation and classification, the genes were aligned to the protein sequence database NR (http://www.ncbi.nlm.nih.gov). NR annotation and Gene ontology (GO) analysis were used to predict gene function, and identify the functional category distribution frequency^69^. GO classifications were obtained according to molecular function, biological process, and cellular component. KEGG annotation (http:www.genome.jp/kegg) was performed to identify the metabolic pathways of genes.

### RNA-seq data validation by quantitative real-time PCR

To validate the genes expression patterns displayed by RNA-seq results, a total of 16 DEGs were randomly selected and analyzed using quantitative real-time reverse transcription PCR (qRT-PCR). Total RNA was extracted from root samples treated by various forms of N using the TRIzol reagent (Invitrogen) according to the manufacturer’s protocol. qRT-PCR amplification was performed using primers designed by Primer 6.0 software for targeted genes as described. Three biological replicates were included. The expression levels of targeted genes were normalized based on the expression levels of *CsACTIN* in different root samples^70^. All the primers for genes amplification using qRT-PCR were listed in the Supplemental Table S11.

## Supplementary Information

Supplementary Information.

Supplementary Table S1.

Supplementary Table S2.

Supplementary Table S3.

Supplementary Table S4.

Supplementary Table S5.

Supplementary Table S6.

Supplementary Table S7.

Supplementary Table S8.

Supplementary Table S9.

Supplementary Table S10.

Supplementary Table S11.

Supplementary Figure S1.

Supplementary Figure S2.

## Data Availability

The datasets analyzed during the current study are available from the corresponding author on reasonable request.
