# Supplementary material for: Transcriptional regulation of amino acid metabolism in response to nitrogen deficiency and nitrogen forms in tea plant root (Camellia sinensis L.)
Source: Sci Rep. 2020 Apr 22;10:6868. doi: 10.1038/s41598-020-63835-6 (PMC7176667; doi:10.1038/s41598-020-63835-6)
Supplement: Supplementary file 1 — Supplementary Information. [file 41598_2020_63835_MOESM1_ESM.docx]

**Supplementary Information**

Transcriptional regulation of amino acid metabolism in response to nitrogen deficiency and nitrogen forms in tea plant root (*Camellia sinensis* L.)

Tianyuan Yang, Huiping Li, Yuling Tai, Chunxia Dong, Enhua Xia, Ziping Chen, Fang Li, Xunmin Cheng, Xiaochun Wan, Zhaoliang Zhang

**Legends of Supplementary tables and figures**

**SUPPLEMENTARY MATERIAL**

**Fig. S1** **Effects of N forms and 0 N on relative fold change of amino acid contents in tea plant root**. The relative fold changes of 15 amino acids were calculated under treatments with four forms of N. Data shown are the average mean ± SE of three replicates (n = 3). Different letters indicate statistical significance among different treatments according to Duncan’s multiple range test at the 5% level.

**Fig. S2 Total N concentration of tea plant roots under various forms of N treatments**. Data shown are the average mean ± SE of three replicates (n = 3). Different letters indicate statistical significance among different treatments according to Duncan’s multiple range test at the 5% level.

**Table S1** Content (mg/g fresh weight) of amino acids in tea plant root under different forms of N treatments and the percentage represented for amino acids contents from their respective pathways in comparison to total amino acids under various form of N.

**Table S2** Quality assessment of the RNA-seq data.

**Table S3** Expression analysis of genes identified in tea roots under different forms of N and 0 N.

**Table S4** DEGs in tea plant roots under treatments with various forms of N.

**Table S5** Co-expressed and uniquely expressed DEGs from tea roots treated WITH different forms of N for 10 days.

**Table S6** Potential genes encoding biosynthetic enzymes as well as enzymes catalyzing the initial amino acid catabolic steps.

**Table S7** Potential genes and DEGs encoding enzymes related to the Glu pathway in tea plant roots under various forms of N.

**Table S8** Potential genes and DEGs encoding enzymes related to the Asp and pyruvate pathways in tea roots under various forms of N.

**Table S9** Potential genes and DEGs encoding enzymes related to the phosphoenolpyruvate pathway in tea plant roots under various forms of N.

**Table S10** Potential genes and DEGs encoding enzymes related to the 3-phosphoglycerate pathway in tea plant roots under various forms of N.

**Table S11** Nucleotide sequences of primers used for qRT-PCR.


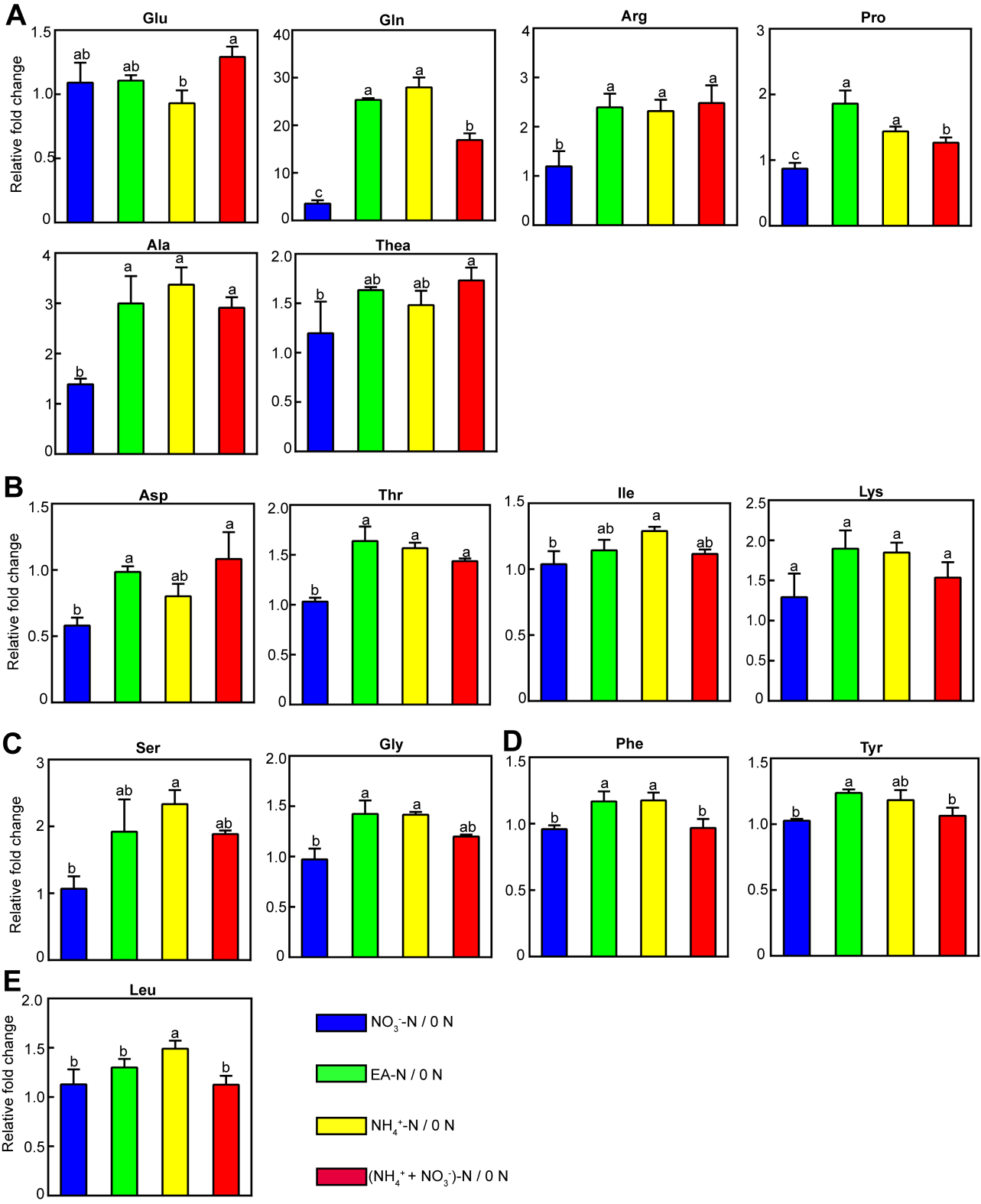


**Fig. S1** Effects of N forms and 0 N on relative fold change of amino acid contents in tea plant root.


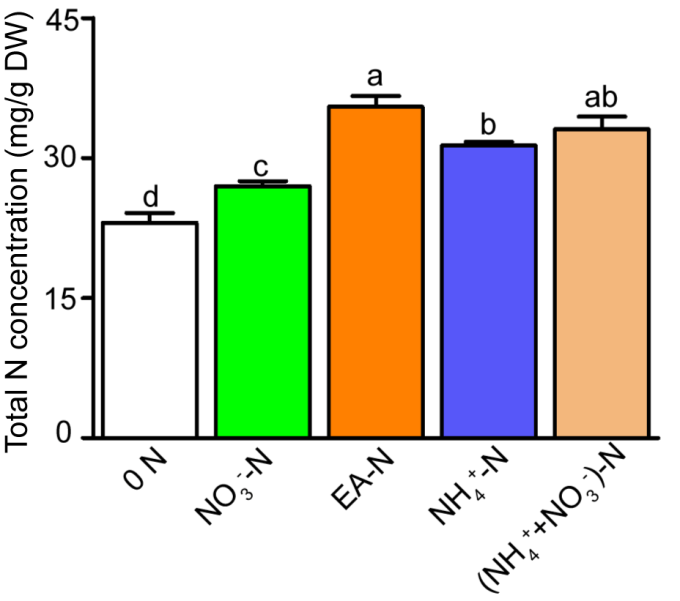


**Fig. S2** Total N concentration of tea plant roots under various forms of N treatments.
