# Supplementary material for: Transcriptional regulation of amino acid metabolism in response to nitrogen deficiency and nitrogen forms in tea plant root (Camellia sinensis L.)
Source: Sci Rep. 2020 Apr 22;10:6868. doi: 10.1038/s41598-020-63835-6 (PMC7176667; doi:10.1038/s41598-020-63835-6)
Supplement: Supplementary file 2 — Supplementary Table S1. [file 41598_2020_63835_MOESM2_ESM.docx]

Table S1 Content (mg/g fresh weight) of amino acids in tea plant roots under different forms of nitrogen treatments and the percentage represented for ralative amino acids content from different pathways in comparison to total amino acids.

| (mg/g FW. Root) |  | 0 N | NO_3_^-^-N | EA-N | NH_4_^+^-N | (NH_4_^+^-NO_3_^-^)-N |  |
| --- | --- | --- | --- | --- | --- | --- | --- |
| Glutamic Acid |  | 0.27± 0.012b | 0.29± 0.012ab | 0.30± 0.023ad | 0.25± 0.028b | 0.35 ± 0.036a | |
| Glutamine |  | 0.034± 0.0017c | 0.12± 0.0078c | 0.85± 0.053a | 0.94± 0.10a | 0.56± 0.020b | |
| Arginine |  | 0.22 ± 0.012b | 0.26± 0.025b | 0.51± 0.033a | 0.50 ± 0.019a | 0.54 ± 0.079a | |
| Theanine |  | 8.76 ± 0.075c | 10.47 ± 1.55bc | 14.32± 0.36a | 12.98 ± 1.23ab | 15.2 ± 1.27a | |
| Proline |  | 0.10 ± 0.0033cd | 0.088 ± 0.0068d | 0.19 ±0.016a | 0.14± 0.0062b | 0.13± 0.0084bc | |
| Alanine |  | 0.020 ± 0.0008b | 0.028 ± 0.0022b | 0.059± 0.009a | 0.068± 0.010a | 0.058 ± 0.0044a | |
| Leucine |  | 0.051 ± 0.0036c | 0.057 ± 0.0013bc | 0.066 ± 0.001ab | 0.076 ±0.004a | 0.057± 0.0056bc | |
| Isoleucine |  | 0.26 ± 0.015b | 0.27±0.004a | 0.30±0.008a | 0.34 ± 0.0098a | 0.29 ± 0.019a | |
| Aspartic Acid |  | 0.21± 0.013a | 0.12±0.0056b | 0.21±0.018a | 0.17±0.019ab | 0.23±0.045a | |
| Threonine |  | 0.69 ± 0.029b | 0.71±0.015b | 1.12± 0.074a | 1.08 ± 0.076a | 0.99 ± 0.047a | |
| Lysine |  | 0.053 ±0.006d | 0.060 ±0.005cd | 0.099±0.0045a | 0.10±0.003a | 0.081 ±0.009bc | |
| Serine |  | 0.10 ±0.0075b | 0.11±0.0058b | 0.19±0.032a | 0.24 ±0.034a | 0.19 ±0.011a | |
| Glycine |  | 0.023 ±0.0012b | 0.022±0.0007b | 0.032±0.0028a | 0.032±0.0024a | 0.027 ±0.001ab | |
| Phenylalanine |  | 0.056±0.0022b | 0.054±0.0013b | 0.065 ±0.0022a | 0.065 ±0.002a | 0.054 ±0.003b | |
| Tyrosine |  | 0.41 ±0.0035b | 0.42±0.0067b | 0.50 ±0.01a | 0.48 ±0.028ab | 0.43 ±0.022b | |
| Total |  | 11.44 ±0.19b | 13.30±1.66b | 19.91 ±0.76a | 18.57 ±1.61a | 20.29±1.68a | |

Values shown are means ± SE (n=3 biological replicates).Different letters within rows indicate a significant difference between the means compared with 0 N treatment according to Duncan’s multiple range test at the 5% level.

| Pathways |  | 0 N (%) | NO_3_^-^-N (%) | EA-N(%) | NH_4_^+^-N (%) | (NH_4_^+^-NO_3_^-^)-N (%) | |
| --- | --- | --- | --- | --- | --- | --- | --- |
| P1(Glu,Gln,Arg,Pro) |  | 5.51± 0.17 | 5.89± 0.66 | 9.85± 0.36 | 10.55± 0.19 | | 8.33± 0.84 |
| P2(Thea) |  | 77.83±0.58 | 79.51± 2.37 | 76.14± 0.47 | 74.21± 0.75 | | 78.97± 2.02 |
| P3(Asp,Thr,Lys,lle) |  | 10.83±0.39 | 9.23± 1.12 | 9.18± 0.27 | 9.73± 0.39 | | 8.42± 0.98 |
| P4(Ala,Leu) |  | 0.63±0.04 | 0.67± 0.08 | 0.66± 0.03 | 0.82± 0.02 | | 0.61± 0.08 |
| P5(Tyr,Phe) |  | 1.10±0.08 | 1.02± 0.15 | 1.16± 0.16 | 1.54± 0.12 | | 1.14± 0.12 |
| P6(Gly,Ser) |  | 4.10±0.05 | 3.68± 0.40 | 3.01± 0.04 | 3.15± 0.26 | | 2.53± 0.09 |
