# Supplementary figures and images for: Transcriptional regulation of amino acid metabolism in response to nitrogen deficiency and nitrogen forms in tea plant root (Camellia sinensis L.)
Source: Sci Rep. 2020 Apr 22;10:6868. doi: 10.1038/s41598-020-63835-6 (PMC7176667; doi:10.1038/s41598-020-63835-6)

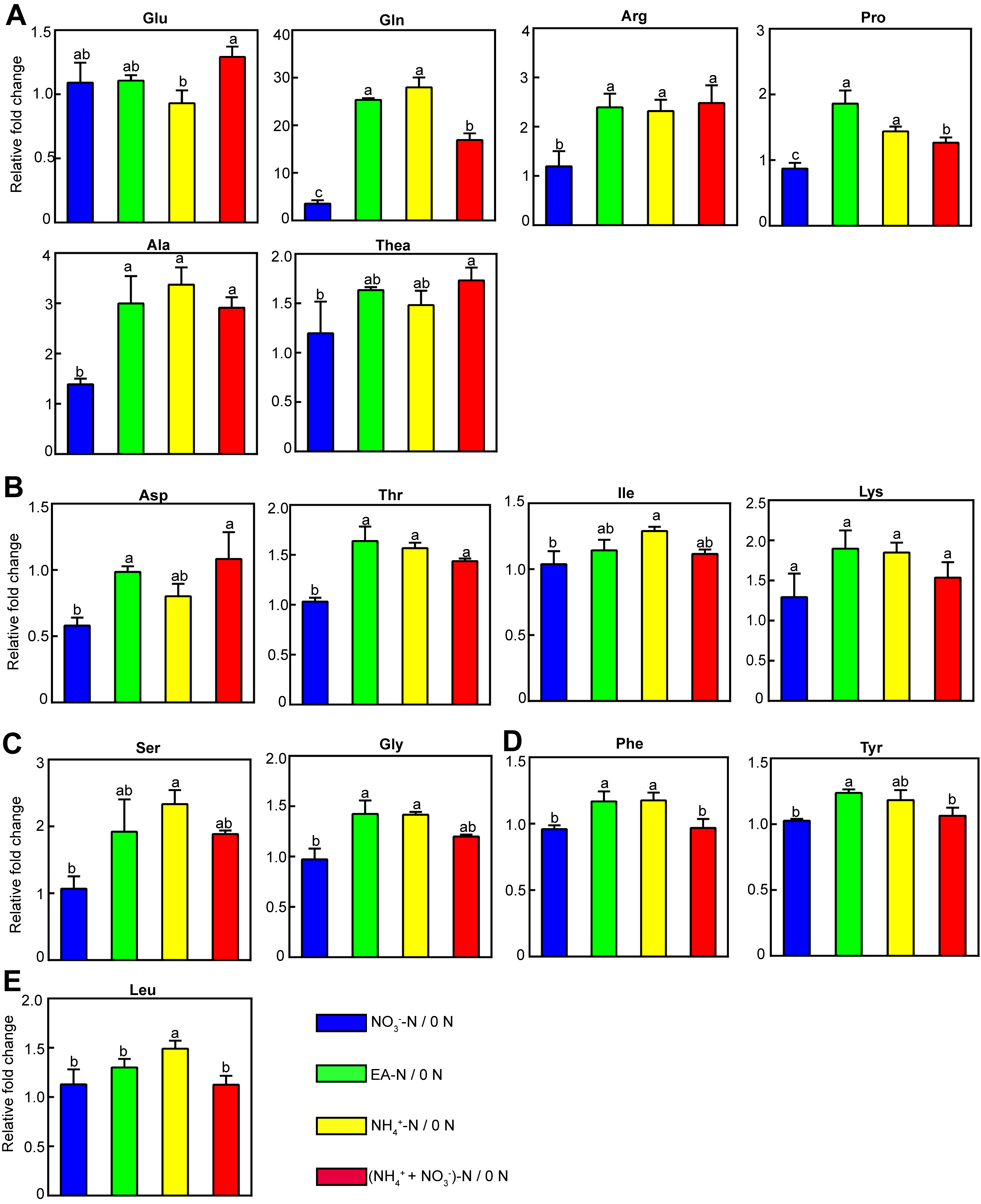

Supplement: Supplementary file 13 — Supplementary Figure S1. [file 41598_2020_63835_MOESM13_ESM.tif]

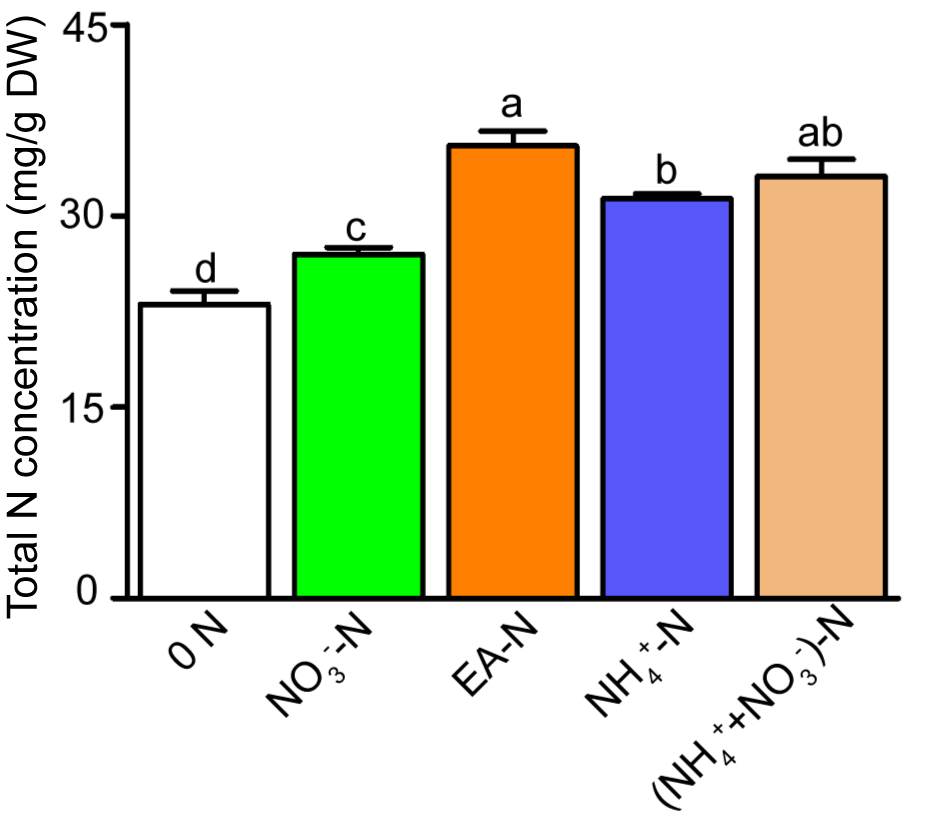

Supplement: Supplementary file 14 — Supplementary Figure S2. [file 41598_2020_63835_MOESM14_ESM.tif]
